# Enhancing mechanical and freeze-thaw performance of MICP-treated sand through palm fiber reinforcement: A sustainable approach for sandy soil stabilization

**DOI:** 10.1371/journal.pone.0332051

**Published:** 2025-09-15

**Authors:** Huan Tao, Chaochao Sun, Jili Qu, Yuandong Huang

**Affiliations:** 1 School of Environment and Architecture, University of Shanghai for Science and Technology, Shanghai, China; 2 School of Civil Engineering, Kashi University, Xinjiang, China; Graphic Era Deemed to be University, INDIA

## Abstract

The integration of palm fiber with Microbially Induced Calcite Precipitation (MICP) technology offers a sustainable and bio-based approach to enhance the mechanical performance and durability of sandy soils, particularly under freeze-thaw conditions. In this study, a systematic experimental investigation examines the effects of varying palm fiber contents (0%−0.30%) on the bearing capacity, crust thickness, calcium carbonate deposition, and freeze-thaw resistance of MICP-treated sand. Results indicate that mechanical performance improves with increasing fiber content, peaking at 0.15%, beyond which the benefits diminish due to fiber agglomeration. At the optimal dosage, the bearing capacity increases by 24%, crust thickness by 70.5%, and calcium carbonate content reaches 16.8% compared to fiber-free MICP samples. Freeze-thaw tests demonstrate higher mass and strength retention, indicating improved durability. Microstructural analyses using SEM, XRD, EDS, and FTIR reveal enhanced microbial attachment and uniform CaCO₃ precipitation along fiber-sand interfaces, which strengthens matrix cohesion. These findings uncover a hybrid bio-mechanical reinforcement mechanism and highlight the trade-offs between fiber dosage and pore connectivity. This study provides novel insights into fiber-assisted biomineralization and offers a viable pathway for environmentally friendly soil reinforcement. Furthermore, potential directions such as predictive modeling, biodegradability assessments, and field-scale application are proposed to support long-term geotechnical and ecological engineering deployment.

## Introduction

Microbially Induced Calcite Precipitation (MICP) is an environmentally friendly soil improvement technique based on biologically induced mineralization. It utilizes urease-producing bacteria, such as *Sporosarcina pasteurii*, to catalyze the hydrolysis of urea. The resulting carbonate ions react with calcium ions in the surrounding medium to form calcium carbonate (CaCO₃), which precipitates and binds soil particles together, thereby enhancing soil strength and reducing permeability [1].

The biochemical reactions involved are outlined as follows:


$$\text{CO}{\left({\text{NH}}_{\text{2}}\right)}_{\text{2}}+{\text{H}}_{\text{2}}\text{O}\to \text{C}{\text{O}}_{\text{2}}+\text{N}{\text{H}}_{\text{3}}$$ (1)

$$\text{C}{\text{O}}_{\text{2}}+{\text{H}}_{\text{2}}\text{O}\to \text{C}{\text{O}}_{\text{3}}^{\text{2}-}+\text{2N}{\text{H}}_{\text{4}}^{+}$$ (2)

$$\text{C}{\text{a}}^{\text{2}+}+\text{C}{\text{O}}_{\text{3}}^{\text{2}-}\to \text{CaC}{\text{O}}_{\text{3}}$$ (3)

MICP has gained attention for its advantages in energy efficiency, carbon emission reduction, and the replacement of conventional cement-based materials. It has shown success in improving soil bearing capacity, resisting erosion, and mitigating sandstorms in arid regions [2–4]. However, previous studies have largely focused on short-term mechanical performance or used synthetic fibers or well-studied natural fibers such as jute, coir, or lignin [5–7]. In contrast, this study introduces palm fiber, a naturally abundant, underutilized lignocellulosic biomass, as a reinforcing additive in the MICP process. Palm fiber offers high tensile strength and surface roughness, which potentially enhance microbial adhesion and calcite nucleation. Unlike prior research which has not addressed its degradation behavior, the present work investigate the long-term stability and biodegradability of palm fiber in a microbially cemented matrix-factors crucial to real-world engineering applications. Moreover, this study applies a comprehensive analytical framework combining mechanical testing (bearing capacity, crust thickness, freeze-thaw resistance) with multi-scale microstructural observations (SEM, EDS, XRD, FTIR), enabling a deep mechanistic understanding of the interactions between palm fiber, microbial activity, and calcium carbonate formation. By bridging the gap between short-term laboratory results and the longer-term environmental sustainability of biocemented soils, our work provides novel insights into the optimization and durability of natural-fiber-enhanced MICP for surface stabilization in harsh climates.

**Fig 1:**
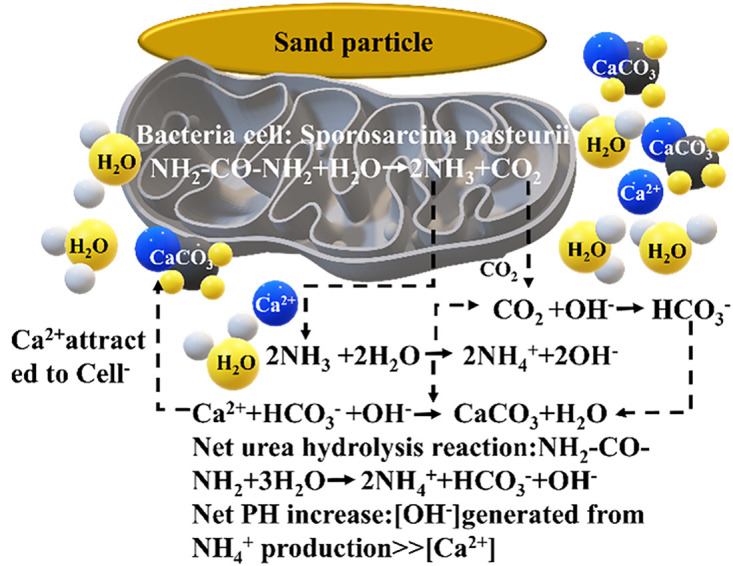
Schematic drawing of procedure of microbial-induced calcite precipitation (MICP).

**Fig 2:**
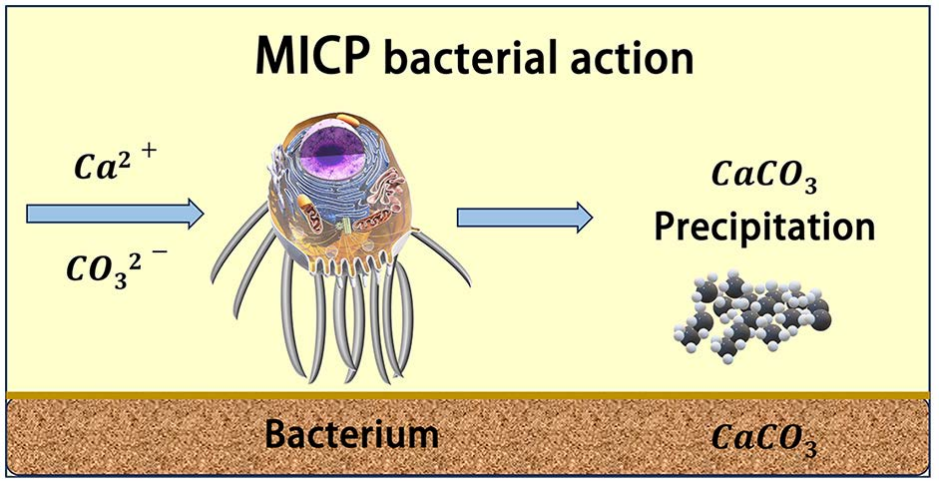
Schematic illustration of microbially induced calcium salt deposition.

This technology is predicated upon the biochemical mineralization process transpiring in the soil naturally. Urea hydrolyzed by urease secreted by microorganisms combines with calcium ions to generate calcium carbonate, cementing soil particles and thereby upgrading the physical and mechanical attributes of the soil. The chemical formula and diagram of the reaction are displayed in Fig 1. The schematic illustration of calcium salt deposition induced by microorganisms is depicted in Fig 2.

MICP technology has attracted considerable scholarly attention owing to its economic feasibility, high efficacy, and environmental compatibility. The investigations carried out by Whiffin [2**]** and Dejong et al. [3] show that MICP technology can conspicuously enhance the strength and stiffness of sand. Nevertheless, the sample typically necessitates multiple cycles of MICP treatment to achieve a superior curing outcome. Simultaneously, Cui et al. [4] disclose that microbial-solidified sand exhibits pronounced brittle failure characteristics, which, to a certain extent, constrains the application of MICP technology in practical geotechnical engineering. Fiber reinforcement technology is an emergent type of soil improvement methodology, which can augment the engineering mechanical properties of soil by uniformly integrating a specific amount of fiber into the soil. Gray et al. [5] incorporate fiber into sand to strengthen the sand and minimize the strength loss after the peak strength. Yetimoglu et al. [6] ascertain through laboratory experiments that fiber reinforcement can increase the residual shear strength of desert sand and ameliorate the shear brittleness failure property of sandy soil by increasing the fiber content.

Shao et al. [7] demonstrate that fiber significantly influences the shear strength of sandy soil, reducing post-peak strength loss and enhancing soil ductility. Collectively, these studies suggest that fiber addition mitigates post-peak strength loss and improves the brittle failure characteristics of soil. Recent research [8,9] confirms that combining fiber reinforcement with microbial solidification technology ameliorates the brittle failure properties of biocemented soil, though only fiber content has been thoroughly examined. Further investigations [10–14] explore fiber-reinforced soil’s dynamic characteristics-including dynamic strength, elastic modulus, and damping ratio—through various dynamic tests, consistently affirming fiber’s reinforcing effect. Existing studies show that MICP-fiber composite modification markedly improves sandy soil’s static properties [15–20]. Additionally, fiber incorporation promotes calcium carbonate precipitation during MICP, while fiber type, length, and dosage substantially influence curing efficacy [21–28].

Through experiments, Lin Shengqiang et al. ascertain that the addition of a specific quantity of carbon fiber significantly enhances both the dynamic strength and deformation resistance of MICP-solidified calcareous sand [29]. Zheng Junjie’s team investigates the influence of fiber reinforcement on microbial-solidified soil and examines the compressive and shear strength of samples with varying fiber contents, demonstrating that fiber reinforcement effectively improves soil strength and softening characteristics [30]. Based on shaking table tests conducted by Maheshwari et al. [31], synthetic fiber and natural coconut shell fiber can notably delay the liquefaction time of sandy soil foundations and reduce foundation settlement under lower acceleration, exerting a significant effect on mitigating liquefaction. Noorzad et al. [32] explore the enhancement of dynamic characteristics in sandy soil of different densities using discrete fibers through dynamic triaxial tests. Their results indicate that the number of liquefaction cycles and the dynamic shear modulus of medium-dense sandy soil increase significantly with higher fiber content and longer fiber length, while fiber reinforcement strengthens the sample’s anti-liquefaction and anti-deformation capabilities.

This paper systematically investigates the effects of varying palm fiber contents (0.05%, 0.15%, 0.25%, and 0.30%) on the bearing capacity and freeze-thaw durability of microbially induced carbonate precipitation (MICP)-treated sandy soil. A comprehensive suite of tests – including bearing capacity measurements, freeze-thaw cycle evaluations, X-ray diffraction (XRD), scanning electron microscopy (SEM), energy dispersive spectroscopy (EDS), and Fourier transform infrared spectroscopy (FTIR)-analyzes the mechanical performance, calcium carbonate formation, and microstructural evolution of the fiber-reinforced samples.The integration of biodegradable palm fibers into MICP treatment introduces a novel reinforcement strategy that enhances crust formation, microbial adhesion, and long-term structural integrity. Unlike prior studies, this research provides a quantitative assessment of crust thickness, CaCO₃ yield, and mechanical stability under cyclic environmental stresses, while also addressing the threshold effects of excessive fiber dosage. Moreover, this work proposes future directions involving predictive modeling and machine learning for parameter optimization, offering new insights into scalable, eco-friendly solutions for geotechnical and environmental engineering applications. Furthermore, this study have summarized a contribution table as shown in Table 1.

**Table 1:** Contribution summary.

Contribution	Description
Integration of natural fiber with MICP	Pioneering use of palm fiber in MICP-treated sands to improve environmental adaptability and sustainability.
Quantitative evaluation of durability	Detailed testing of bearing capacity, crust thickness, CaCO₃ yield, and freeze–thaw cycles under various fiber dosages.
Microstructural mechanism elucidation	SEM, FTIR, XRD, and EDS analyses provide insights into the interaction between fiber, sand, and microbial precipitates.
Innovation in predictive modeling	Outlines potential use of ANN, SVM, and genetic algorithms for optimizing MICP parameters.
Engineering application insights	Discussion of scalability, environmental implications, and challenges in field deployment.

## Test materials and experimental procedures

### Test material

#### Sand.

**Fig 3:**
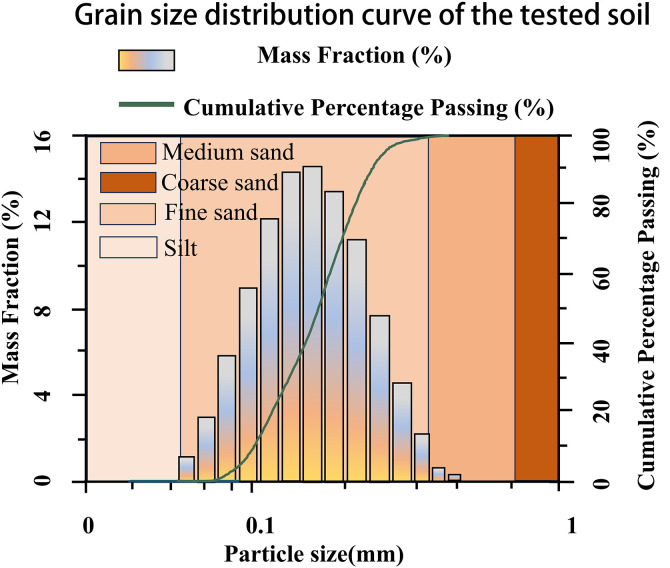
Grain size distribution curve of the tested soil. Y-axis represents the cumulative percentage of particles passing through the sieves.

The siliceous sand used in this study originates from a desert area near Kashi Meghati County (77° 22’49“E, 39°1’11”N). Sample collection occurs in a non-protected area with minimal environmental disturbance, complying with local academic research regulations that require no special license. Fig 3 displays the grain size distribution of the sand [33–35], and Table 2 summarizes its physical and chemical properties.The desert sand samples utilized in this study originate exclusively from areas designated for scientific research sampling, as permitted by local authorities. The sampling process strictly complies with regulations established by local government and administrative departments. This research requires no specific license, as the collection of small quantities of desert sand for scientific purposes is exempt under applicable laws. 1. Non-Destructive and Small-Scale Scientific Research Collection

**Table 2:** Physical and chemical properties of original desert soil.

Name	Moisture content (%)	Dry density (g/cm^3^)	Void ratio	Specific Gravity	Effective particle size_10_	D_10_	D_30_	D_60_	C_U_	C_C_	PH value
Desert soil	0.7	1.58	0.758	2.68	0.086	0.2	0.3	0.44	2.18	1.06	8.76

In accordance with the “Regulations of the People’s Republic of China on Nature Reserves” (revised in 2017) and the “Mineral Resources Law of the People’s Republic of China,” if the collection activity satisfies the following criteria, no additional permission is required:

This study employs minimal collection volumes, using only negligible quantities of sand and soil exclusively for scientific research purposes without commercial intent. The collection process maintains strict environmental safeguards, ensuring: (1) no damage to surface vegetation, (2) preservation of geological structures, and (3) elimination of adverse ecological impacts through controlled sampling protocols.2. Non-Protected Areas:

The desert sand used in this study originates from areas located outside all protected zones, including nature reserves, national parks, ecological red lines, mineral resource reserves, farmland, and other ecologically sensitive areas. Sample collection strictly adheres to environmental protection requirements, ensuring compliance with all relevant regulations governing scientific research activities.

Grain size distribution curve of the tested soil. According to the Unified Soil Classification System (USCS) and ASTM D2487-17 [33] standards, the soil is classified as poorly graded silty sand (SP-SM), based on the predominance of sand-sized particles and limited fine content (8.7% < 0.075 mm).

According to the Unified Soil Classification System (USCS) and ASTM D2487-17 [33] standards, the soil is classified as poorly graded silty sand (SP-SM), based on the predominance of sand-sized particles and limited fine content (8.7% < 0.075 mm). Cu = 2.18 and Cc = 1.06 reflect its narrow gradation.

The grain size distribution of the soil is determined in accordance with ASTM C136/ C136M-19 [34]/ ASTM D6913/ D6913M-17 [35] and classified using the Unified Soil Classification System (USCS) and ASTM D2487-17 [33]. The results indicate that only 8.7% of the particles are finer than 0.075 mm, which does not meet the criteria for silt or clay classification (≥50%). Instead, the soil is categorized as poorly graded silty sand (SP-SM) due to the dominance of sand-sized particles and relatively low gradation. The uniformity coefficient (Cu) and curvature coefficient (Cc) are calculated as 2.18 and 1.06, respectively, which align with the characteristics of narrowly graded sandy soils with minor fines.

#### Palm fiber.

**Fig 4:**
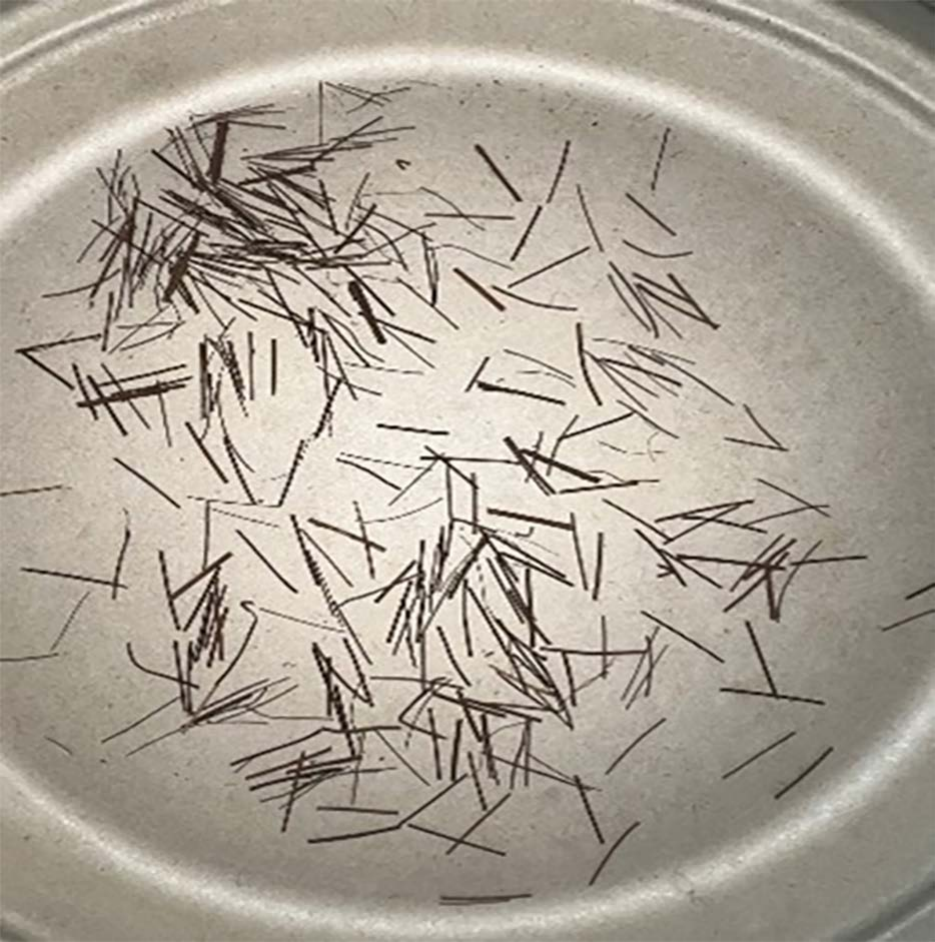
Physical morphology of raw palm fiber before mixing, showing typical yellowish-brown surface texture and fibrous bundle structure.

The palm fiber used in the test is shown in Fig 4.

**Palm Fiber Pretreatment:** In the experimental procedure, palm fibers undergo washing followed by drying at 80°C for 24 hours. Subsequent cutting processes produce uniform 12 mm lengths to ensure homogeneous distribution throughout the mixture. Researchers record moisture content and physical properties immediately before mixing to establish baseline material characteristics.Sand-Fiber Mixing:

The experimental protocol involves thoroughly mixing pretreated fibers with dry silica sand at mass fractions of 0%, 0.05%, 0.10%, 0.15%, 0.20%, 0.25%, and 0.30%. A mechanical mixer ensures uniform dispersion through continuous blending for 10 minutes. Mechanical Performance:

While synthetic fibers (e.g., polypropylene, polyethylene) generally exhibit higher tensile strength and chemical resistance, palm fibers-when combined with MICP--achieve comparable improvements in compressive strength and ductility due to the effective calcium carbonate bridging and microbial attachment sites enhanced by their rough surface morphology. Literature reports suggest that MICP-palm fiber composites can achieve strength gains of 20–35%, which is within the range reported for synthetic fiber-soil systems under low dosage conditions.

**Economic and Environmental Benefits:** Palm fibers are agricultural by-products, readily available in tropical and subtropical regions, and are significantly cheaper than industrial synthetic fibers. Moreover, they are biodegradable and renewable, unlike synthetic alternatives which may cause microplastic pollution. Geopolymers, while mechanically robust and durable, often rely on high-purity aluminosilicates and energy-intensive alkaline activators, which can elevate both costs and carbon footprints.

**Field Scalability and Biocompatibility:** The integration of palm fibers into MICP-treated sand demonstrates high biocompatibility with microbial activity, whereas certain synthetic polymers may inhibit microbial urease efficiency. Palm fibers also facilitate microbial colonization and uniform CaCO₃ deposition, a feature not inherent to synthetic reinforcements [20,36].

The physical and chemical characteristics of palm fiber are summarized in the Table 3 that follows.

**Table 3:** Physical and mechanical properties of palm fiber used in this study.

Palm fiber density (g/cm^3^)	Calorific value (Kcal/kg)	Moisture(%)	Diameter(µm)	Ash content (%)	Impurity content (%)	Modulus of elasticity (MPa)	Tensile strength (MPa)	Elongation at break(%)
1.28	4000	12-30	0.2-0.3	3	0.8	800-1900	87-166	19.0-21.0

Ash content and moisture content are determined experimentally in our laboratory in accordance with ASTM D3174 [37] (Standard Test Method for Ash in Biomass) and ASTM D4442 [38] (Standard Test Methods for Moisture Content of Wood-Based Materials).

Tensile strength and elongation at break values are obtained from literature reports [39–41], as these tests require specialized fiber testing systems which are not available in our current experimental setup.

A 12 mm palm fiber length demonstrates optimal enhancement of both peak stress and residual stress in quartz sand [42], and is therefore selected for this study. The investigation incorporates palm fiber mass fractions of 0%, 0.05%, 0.15%, 0.25%, and 0.30% into siliceous sand samples. For each fiber-reinforced MICP specimen, parallel samples are prepared and their mean values calculated to establish final measurements. This replicate sampling approach ensures experimental reliability and result reproducibility.

**Fig 5:**
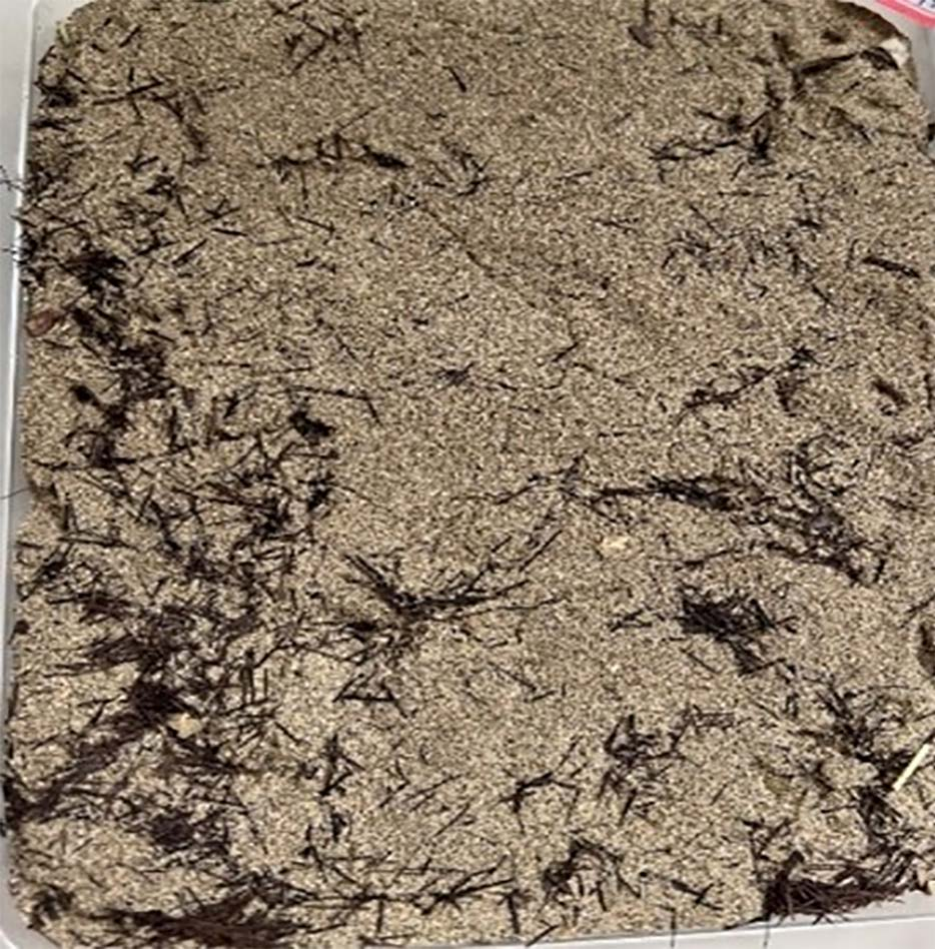
Surface appearance of MICP-treated sand sample after 7-day curing with 0.15% palm fiber content, demonstrating crust formation and structural integrity.

The experimental setup utilizes a cuboid plastic box measuring 25 cm (length) × 18 cm (width) × 2.5 cm (height) (Fig 5). This study establishes six sample groups, including two control groups: (1) undisturbed sand and (2) sand treated with cementation solution only. For each group, three parallel tests ensure statistical reliability, resulting in a total of 18 samples. The testing protocol maintains a 30-day observation period for all specimens.

#### Cementation solution.

The cementation solution consists of yeast extract (0.1 g/L), ammonium chloride (12.5 mM), sodium acetate (42.5 mM), urea (350 mM), and calcium chloride (250 mM) at pH 8.4 [43]. A spray volume of 30 mL [44] optimally stimulates indigenous microorganisms in the sandy soil while providing necessary nutrients and calcium ions for the MICP reaction.

Preliminary laboratory trials demonstrate that this concentration achieves three key outcomes: (1) sufficient CaCO₃ precipitation, (2) prevention of solution overflow, and (3) avoidance of clogging or incomplete mineralization. Higher concentrations risk rapid precipitation and pore clogging, while lower concentrations significantly reduce soil reinforcement effectiveness.

#### Operation procedure.

The palm fiber is homogeneously blended into the siliceous sand according to the specified mass fractions (0%, 0.05%, 0.15%, 0.25%, and 0.30%). Prior to mixing, the fiber is manually combed and air-dried to eliminate agglomeration and ensure uniform dispersion. The composite mixture is then placed into a cuboid plastic mold, and the surface is leveled. A cementation solution-comprising urea (350 mM), calcium chloride (250 mM), sodium acetate, ammonium chloride, and yeast extract, adjusted to pH 8.4-is evenly sprayed onto the sample surface using a calibrated sprayer. The total spraying duration is limited to within 5 minutes to ensure consistent reactivity across specimens. After spraying, all samples are covered with breathable plastic wrap to minimize evaporation and are cured in a constant-temperature chamber (25°C) for a period of 30 days. These steps, including fiber pre-treatment, solution composition control, standardized spraying duration, and consistent curing conditions, are strictly followed to ensure the reproducibility and comparability of experimental results.

To improve clarity and reproducibility, this study have added a new table (Table 4) summarizing the composition, palm fiber content, and group labeling of all 18 desert sand used in the study. This includes the untreated control group, the MICP-only group, and the MICP groups with varying palm fiber dosages (0.05% to 0.25%, in 0.05% increments), each tested in triplicate.

**Table 4:** Composition and labeling of desert sand.

Sample Group ID	Treatment Type	Palm Fiber Content (% by weight)	MICP Applied	Number of Replicates
CK-1, CK-2, CK-3	Control (Untreated)	0.00	No	3
MICP-1, MICP-2, MICP-3	MICP only	0.00	Yes	3
PF-005-1 to PF-005–3	MICP + Palm Fiber	0.05	Yes	3
PF-010–1 to PF-010–3	MICP + Palm Fiber	0.10	Yes	3
PF-015-1 to PF-015–3	MICP + Palm Fiber	0.15 (Optimal dosage)	Yes	3
PF-020–1 to PF-020–3	MICP + Palm Fiber	0.20	Yes	3
PF-025-1 to PF-025–3	MICP + Palm Fiber	0.25	Yes	3

Note: Each group issubjected to the same curing protocol. Palm fiber content is expressed as a mass fraction of dry sand weight.

To ensure accurate interpretation, untreated siliceous sand and palm fiber-alone samples are used as control groups for all spectroscopic and microscopic analyses. These control groups enable precise identification of MICP-induced modifications by establishing critical baselines for comparative analysis across multiple characterization techniques: (1) FTIR analysis detects emerging carbonate absorption bands, (2) XRD reveals new calcite diffraction peaks, and (3) SEM imaging identifies surface mineral deposits and biofilm structures.

### Experimental procedures bearing capacity test

**Fig 6:**
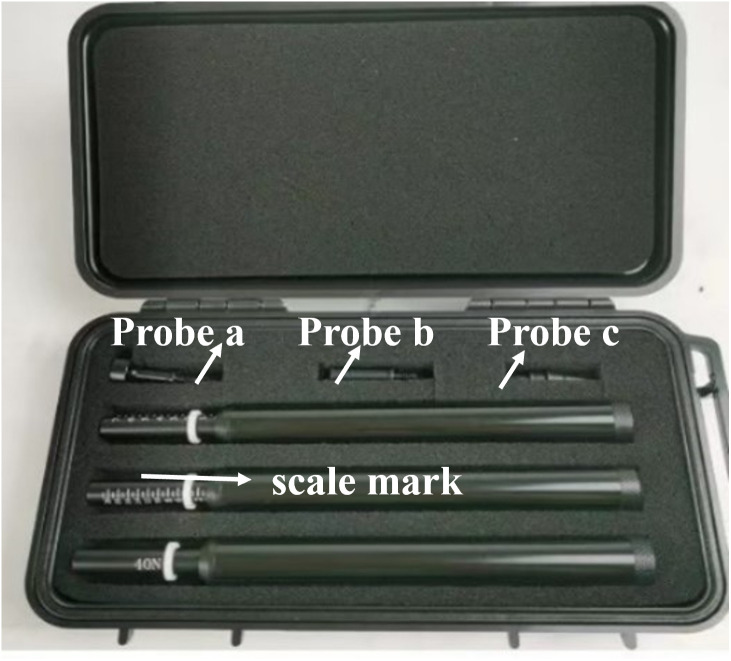
Micro-penetration test device used to evaluate bearing capacity-Measure the soil penetration resistance.

In this paper, a micro-penetration instrument (Fig 6) is employed to evaluate the penetration resistance of the microbial solidified sand, which is subsequently converted into the bearing capacity of the material through a calibrated relationship. This method offers a rapid and reliable assessment of the load-bearing performance of fiber-reinforced MICP-treated specimens.

#### Sample preparation.

Prior to testing, the specimen is cured for a period of 30 days under controlled temperature (25 ± 1 °C). The topmost 2–3 mm loose crust is carefully scraped off using a blade to expose a consolidated and level testing surface. This ensures consistent contact between the probe and the stabilized soil matrix, minimizing measurement deviation caused by surface heterogeneity.

Penetration resistance is measured using a calibrated micro-penetrometer with a 10 mm conical tip (60°angle) at 1 mm/s constant rate, recording force at 10 Hz resolution.

The penetration test is conducted vertically downward with a steady and continuous loading rate. The instrument automatically recorded the penetration resistance (Pt) values (unit: 100 kPa) along the depth profile.

#### Testing procedure.

Position the micro-penetration instrument perpendicularly on the prepared sample surface.

Select and install the appropriate probe according to the soil density and expected resistance.

Initiate the test, allowing the probe to gradually penetrate the soil at a controlled speed.

Continuously record the resistance force as a function of depth using the instrument’s digital acquisition system.

Repeat the measurement at three different points per sample to ensure reproducibility.

#### Calculation of bearing capacity.

The final Pt value at the target depth (20 mm) is used to estimate the bearing capacity (Rpt) by referencing a calibration table provided by the instrument manufacturer [45]. The relationship between Pt and Rpt is established through pre-calibrated empirical fitting using known load-bearing samples, and typically follows the form:


Rpt=α·Pt+β (4)

where:

α and β are calibration constants determined through prior uniaxial compression comparison tests.

#### Quality control.

All tests are conducted at ambient indoor conditions (20–25 °C, RH 60–70%).

To minimize operator bias, a single trained technician performed all measurements.

Results with deviation exceeding ±10% from the average are discarded and re-tested.

### Crust thickness measurement

Following the bearing capacity tests, the crust thickness of each sample is measured to evaluate the structural integrity of the MICP-solidified surface layer [46–48]. The thickness of the hardened crust is gauged at five locations using a precision digital vernier caliper-namely, the four corners and the central area of the specimen’s top surface.

#### Measurement steps.

Use a clean blade to gently remove any loose particles or uneven surface residue from the sample.

At each designated position (four corners and one center), place the vernier caliper perpendicularly to the surface and record the vertical distance from the sample surface to the base of the crust.

Record the five values and compute their average, denoted as $D_{1}$.

#### Repeated group testing.

For each fiber content group, three identical samples are tested under the same curing and MICP conditions.

The average crust thickness values for the three replicates are recorded as $D_{1}$, $D_{2}$ and $D_{3}$.

The final result is calculated as the mean crust thickness across all three trials using the following equation:


$$D=\frac{D_{1}+D_{2}+D_{3}}{3}$$ (5)

This approach ensures result consistency by minimizing sample heterogeneity effects.

#### Instrument parameters.

Instrument: Digital Vernier Caliper
Resolution: 0.01 mm
Accuracy: ± 0.02 mm
Measuring range: 0–150 mm
Calibration: Performed weekly using a gauge block

A summary of the caliper specifications is provided in Table 5.

**Table 5:** The parameters of the vernier calipers.

Name	Resolution(mm)	Measuring range(mm)
Vernier calipers	0.02	150

### Calcium carbonate content test

The quantification of calcium carbonate generated in MICP-treated specimens is performed using the ethylenediaminetetraacetic acid (EDTA) complexometric titration method, which is widely adopted in soil stabilization research [49]. This method enables accurate evaluation of the calcium carbonate mass fraction (W%) based on the chemical consumption of EDTA required to chelate calcium ions in the dissolved sample.

#### Principle.

The calcium carbonate in the solidified sand is dissolved via acid digestion, releasing calcium ions (Ca²⁺), which are then titrated with a standard EDTA solution. The difference in EDTA consumption between the sample and the blank reflects the amount of calcium carbonate present.

#### Formula for calculation.

The calcium carbonate content W% is calculated using the following equation [50]:


$$W\%=\frac{C\times \left(V-V_{0}\right)\times 0.1001}{m}\times 100$$ (6)

Where:

C: Molar concentration of the EDTA standard titration solution (mol/L)

V: Volume of EDTA solution consumed in sample titration (mL)

$V_{0}$: Volume of EDTA solution consumed in the blank test (mL)

m: Mass of the sample (g)

0.1001: The mass (in grams) of calcium carbonate equivalent to 1.000 mL of 1.000 mol/L EDTA solution

#### Experimental steps.

Accurately weigh approximately 10 g of the oven-dried crushed sample and place it in a beaker.

Add excess dilute hydrochloric acid (e.g., 1 mol/L HCl) to dissolve calcium carbonate.

Filter the residue and collect the filtrate.

Titrate the filtrate with standardized EDTA solution using Eriochrome Black T as the indicator.

Record the titrant volume V, and perform a blank titration under the same conditions to obtain $V_{0}$.

Calculate the calcium carbonate content for each sample using Equation (6).

#### Result processing.

Each test group consisted of three parallel determinations. The final calcium carbonate content isreported as the arithmetic mean of the three results to enhance measurement reliability and reduce experimental error.


$$W\%=\frac{C\times \left(V-V_{0}\right)\times 0.1001}{m}\times 100$$ (7)

### Freeze-thaw cycle test

**Fig 7:**
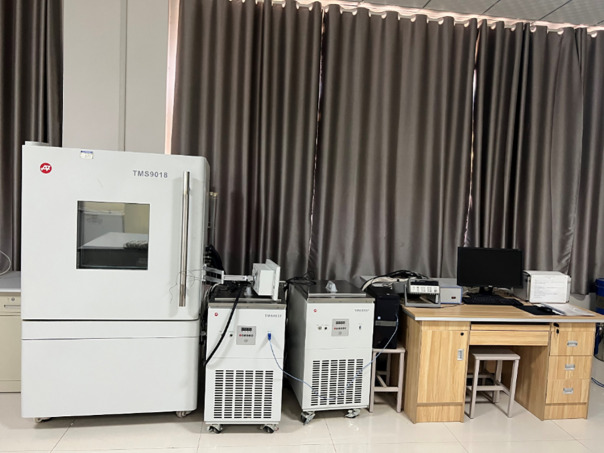
The instrument of freeze-thaw test.

The freeze-thaw durability of MICP-solidified palm fiber-reinforced sand specimens is assessed using a programmable TMS 9018-800S freeze-thaw testing chamber, as depicted in Fig 7. This evaluation simulates the environmental stresses caused by cyclic seasonal temperature fluctuations, which are critical for determining the long-term performance of biomineralized soils.

#### Climatic context.

According to regional meteorological records over the past three decades:

The average minimum temperature in winter is approximately –15 °C;
The extreme minimum temperature reaches –20 °C;
The average summer temperature is approximately 30 °C.
These climatic conditions are taken into consideration to define the freeze-thaw testing regime, ensuring realistic and regionally relevant simulation.

**Testing Standard:** The test procedure is performed in strict accordance with the Test Regulations for Rock of Hydropower Engineering (DL/T 5368−2007) [51], which provides standardized guidelines for thermal durability testing of geomaterials.

#### Freeze-thaw parameters.

Freezing temperature: –20 °C
Thawing temperature: + 30 °C
Cycle duration: 48 hours total per cycle (24 h freezing + 24 h thawing)
Number of cycles: 5
Moisture condition: Samples are maintained in a saturated state throughout the test
Each cycle involved continuous exposure to freezing conditions for 24 hours, immediately followed by thawing conditions for another 24 hours. During the process, samples are placed on a perforated steel rack inside the chamber to allow uniform heat exchange.

#### Post-test evaluation.

After completion of five freeze-thaw cycles, samples are removed and dried at 60 °C for 48 hours. Physical degradation (e.g., cracking, spalling) is recorded, and residual bearing capacity and crust thickness are re-measured to assess performance retention.

### Analytical methods

To investigate the microstructural and compositional evolution of sandy soil following MICP treatment, a series of advanced analytical instruments are employed. These instruments facilitated surface morphology observation, element distribution analysis, and the identification of functional groups and chemical bonds in biomineralized products.

Microstructural and compositional analyses were conducted using field-emission scanning electron microscopy (SEM) with energy-dispersive X-ray spectroscopy (EDS), X-ray diffraction (XRD) with Cu-Kα radiation (2θ range: 5–70°), and Fourier-transform infrared spectroscopy (FTIR) in the 400–4000 cm ⁻ ¹ range. These techniques enabled comprehensive characterization of surface morphology, elemental distribution, crystalline phases (particularly calcite identification), and organic-inorganic interactions in the MICP-reinforced composite system. All instruments were calibrated following standard protocols prior to analysis.

## Results of mechanical performance

### Analysis of bearing capacity test results

**Fig 8:**
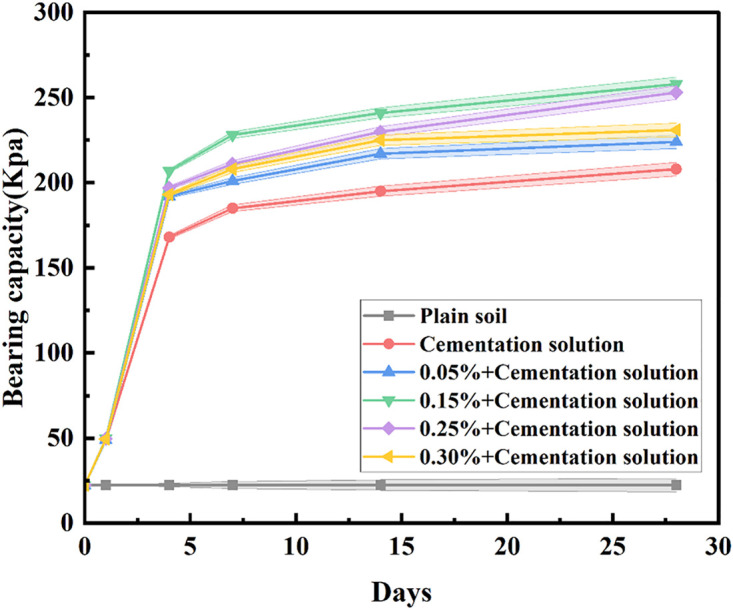
Bearing capacity.

As can be discerned from Fig 8. In this test, on the 28th day of the test, the bearing capacity strength of the undisturbed sand sample was 22.4 kPa, which remains unchanged, whereas that of the cemented liquid sand sample was 208 kPa. Under the circumstance of adding the same amount of cementation solution, when the content of palm fiber is 0.05%, the bearing capacity strength of the sample amounts to 224 kPa; when the content of palm fiber is 0.25%, the bearing capacity strength of the sample reaches 253 kPa; when the content of palm fiber is 0.30%, the bearing capacity strength of the sample is 224 kPa. The bearing strength of the sample is 231 kPa. When the content of palm fiber is 0.15%, the bearing strength attains the peak value of 258 kPa. With the escalation of the mass reinforcement rate, the bearing capacity strength of palm fiber reinforced solidified sand soil initially rises and subsequently drops, which is in accordance with the conclusions in the literature [19,26,29,30,36,52–56]. The frictional and bonding forces existing at the interface between palm fiber and sand particles, together with the spatial restraint effect exerted by palm fiber on sand particles, constrain the displacement of sand particles and enhance the integrity and strength of sand particles.

### Analysis of the fitting function

A nonlinear fitting function was employed to model the relationship between load-bearing capacity and palm fiber content. The fitted curve effectively captures the nonlinear variation trend between the two variables. Based on the significance tests of the fitting parameters and goodness-of-fit indicators such as the coefficient of determination ($R^{2}$) and residual analysis, the results demonstrate that the selected model provides a high fitting accuracy and accurately describes the influence pattern of fiber content on load-bearing capacity. The fitting curve reveals that the load-bearing capacity increases initially and then decreases with increasing fiber content, indicating the existence of an optimal fiber content range that significantly enhances mechanical performance. Beyond this range, the mechanical properties tend to deteriorate. This fitting model offers a theoretical basis for the subsequent optimization of material proportions.

Quadratic Fit function:


$$y=-5471.62\cdot x^{2}+2085.09\cdot x+55.57$$ (8)

Where:

ybearing capacity (kPa)

xPalm fiber content (mass fraction)

Goodness of fit:

$R^{2}=0.817$This indicates that the quadratic function fits the experimental data well and provides a high level of explanatory power.

The bearing capacity of the MICP-treated sand samples initially increased with the addition of palm fiber and peaked at a content of 0.15%, followed by a slight decline at higher fiber contents. This behavior is attributed to two competing mechanisms. At low fiber content (≤0.15%), palm fibers are uniformly distributed, forming a spatial skeleton that effectively restrains sand particle displacement, enhances microbial colonization, and facilitates calcium carbonate deposition. As the content increases beyond this point, fiber agglomeration becomes more prominent, reducing permeability and creating inhomogeneous reaction zones, thereby limiting the reinforcement effect. These observations are consistent with prior studies. Choi et al. [8] demonstrated that fiber incorporation enhances microbial anchoring, which promotes calcite precipitation. Similarly, Fang et al. [49] reported that excessive fiber content may hinder MICP reactions due to clogging effects and uneven bacterial distribution.

In the palm fiber-reinforced sandy soil, the palm-sand interface force mainly originates from the interaction between the surface of palm fiber and sand particles. This is because the interaction between the palm fiber and sand particles dispersed within the sand can restrain the relative sliding of the palm fiber, enabling the palm fiber to bear external loads and transfer loads and reducing the stress concentration in the sand. Furthermore, the palm fibers can bond with each other to form a network, creating a three-dimensional network structure among the sand particles, which restricts the deformation or displacement of the sand particles and thereby enhances the mechanical strength of the desert sand. When the content of palm fiber is low, it is arduous for palm fiber to form webs, which limits the contribution of reinforcement to strength. On the contrary, when the content of palm fiber is overly high, palm fiber is prone to forming clusters during the mixing process, and the reinforcement effect is weakened [56]. Therefore, the appropriate content of 0.15% of palm fiber obtained in this paper can optimize the reinforcement effect of palm fiber, which conforms to the conclusions in the literature [19]. Based on the test, the application of MICP combined with palm fiber reinforcement technology for enhancing the mechanical properties of sand can theoretically augment the strength of the sample, enhance the toughness and improve the mechanical properties of sand.

### Analysis of crust thickness measurement results

**Fig 9:**
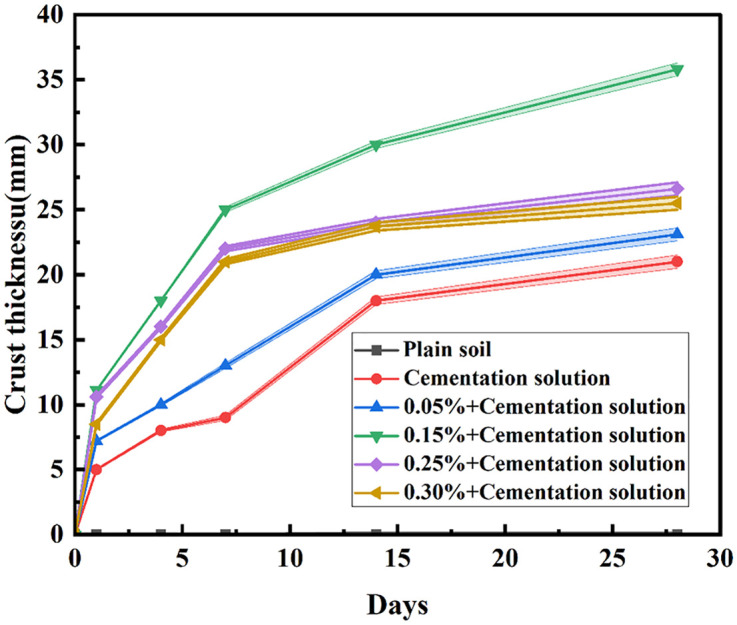
Crust thickness.

It is discernible from Fig 9. that subsequent to the test, the crust thickness of the undisturbed sand sample amounts to 0 mm, whereas that of the cemented liquid sand sample stands at 21 mm. Under the circumstance of adding an identical quantity of cementation solution, when the content of palm fiber is 0.05%, the sample crust thickness is 23.1 mm; when the content of palm fiber is 0.25%, the sample crust thickness is 26.6 mm; when the content of palm fiber is 0.30%, the sample crust thickness is 23.1 mm. The crust thickness of the sample is 25.5 mm, and when the content of palm fiber is 0.15%, the crust thickness is higher, attaining 35.8 mm. The crust thickness ascends with the augmentation of palm fiber content until it reaches the peak value, beyond which it declines with the continued increase of palm fiber content. This is because prior to the crust thickness attaining its peak, during the mixing procedure, the palm fiber will disperse, and the dense fiber filaments offer a propitious adhesion point for bacteria [17,26], augmenting the amount of microbial adhesion and facilitating them to generate more calcium carbonate precipitates during the reaction.

Crust thickness followed a similar trend, increasing with palm fiber content up to 0.15% and then decreasing. This trend highlights the dual role of palm fiber: as a physical scaffold that supports microbial colonization and as a spatial modifier affecting fluid and nutrient transport. The enhancement in crust thickness at optimal fiber content is explained by the increase in available surface area for microbial adhesion and subsequent calcite precipitation. However, at higher fiber concentrations, fiber-fiber contact and entanglement block pore spaces and hinder the penetration of cementation solution, thus leading to localized precipitation and uneven crust formation. Comparable findings have been reported by Yin et al. [17] and Xiao et al. [22].

Nevertheless, when the crust thickness reaches the peak, the likely reason is that the uneven distribution of palm fiber gives rise to the uneven distribution of calcium carbonate within the sample, and the local curing disparity is overly large, resulting in an unsatisfactory crust thickness. When the content of palm fiber attains a certain level, the palm fiber will come into contact and interweave to form a spatial network, which will impede the normal infiltration and migration of microorganisms in the sample [28]. Additionally, palm fiber occupies a considerable amount of pore space, and the limited pore space will restrain the metabolic activities of microorganisms and exert an influence on the generation of calcium carbonate [19]. Furthermore, the pores in the vicinity of the sample surface tend to diminish rapidly, which precludes the cementation solution from permeating into the sand for the MICP process. Hence, the higher the fiber content is not always the better. In this study, the optimal fiber content is 0.15%.

### Calcium carbonate content analysis

The calcium carbonate formed during the MICP process possesses filling and cementing effects, and the production of calcium carbonate is the key factor influencing the reinforcement effect of MICP. Table 6 presents the amount of calcium carbonate generated in samples with different fiber contents after MICP treatment. Within the scope of this study, the calcium carbonate yield of the sample initially increases and subsequently decreases with the increase in the content of palm fiber. When the content of palm fiber rises from 0 to 0.05%, the content of calcium carbonate increases by 33.98%. When the content of palm fiber increases from 0.05% to 0.15%, the content of calcium carbonate is 16.8%, only increasing by 21.74%, and the content of calcium carbonate reaches its peak. When the content of palm fiber increases to 0.25%, the calcium carbonate production decreases by 5.66%, and when the content of palm fiber is 30%, the calcium carbonate content decreases by 8.9%. It can be observed that the relationship between the calcium carbonate production and the content of palm fiber is not a straightforward linear one, which is related to the interaction among microorganisms, palm fiber, and sand particles. It is important to highlight that both the calcium carbonate content and the crust thickness of the undisturbed sand sample are zero, indicating the absence of calcification and crust formation in this particular sample.

**Table 6:** The calcium carbonate content and the crust thickness of the sample.

Name	Crust thickness (mm)	Calcium carbonate content (%)
Undisturbed sand	0	0
Cementation solution	21	10.3
0.05% + Cementation solution	23.1	13.8
0.15% + Cementation solution	35.8	16.8
0.25% + Cementation solution	26.6	15.9
0.30% + Cementation solution	25.5	14.6

The calcium carbonate content increased with fiber addition up to 0.15%, reaching a peak value of 16.8%, then declined as fiber content rose further. This non-linear trend reflects the interaction among microorganisms, palm fibers, and sand particles. At optimal content, the palm fibers act as colonization scaffolds for bacteria, enhancing their proliferation and subsequent urease activity, which increases local carbonate ion production and facilitates nucleation of calcite. However, excessive fiber content reduces the effective pore volume for microbial metabolism and mineral precipitation due to spatial obstruction. This trend is consistent with observations by Zhao et al. [20] and Li et al. [28].

**Fig 10:**
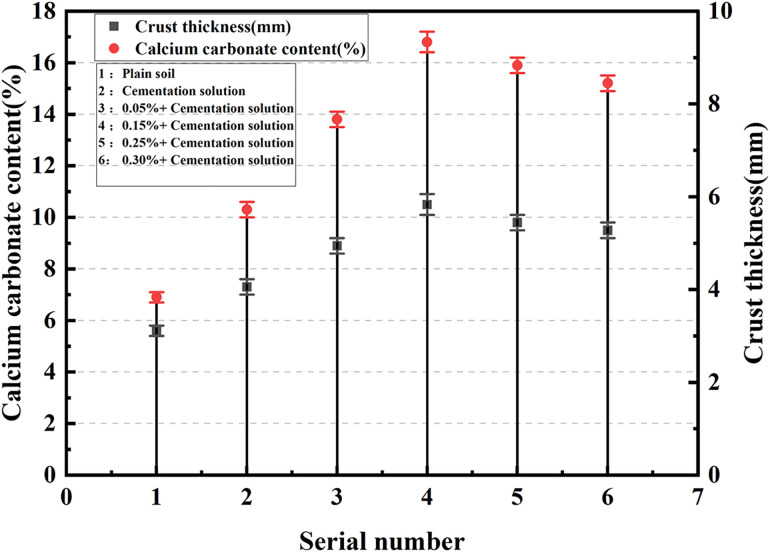
Crust thickness and calcium carbonate content.

**Fig 11:**
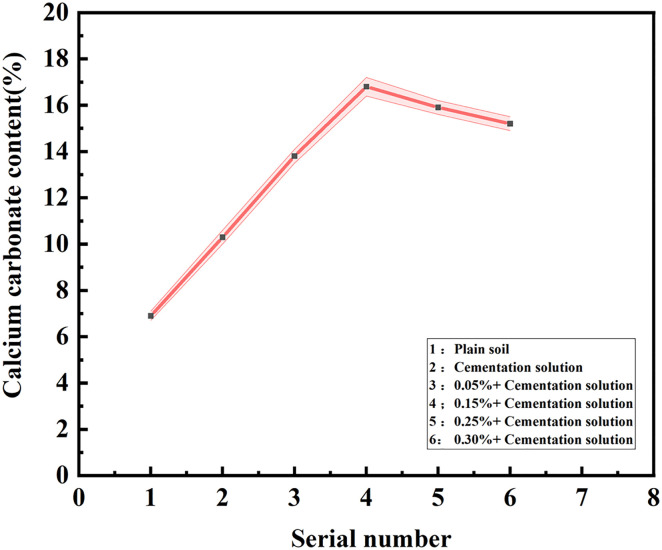
The relationship between calcium carbonate content and cementation solution concentration.

The calcium carbonate content is depicted in Figs 10 and 11. Excluding the undisturbed sand, the sample only the cementation solution was added, without any other additives or treatments possesses the lowest average calcium carbonate content. whereas the sample with 0.15% palm fiber content holds the highest average calcium carbonate content. The calcium carbonate content in all palm fiber-reinforced sand samples is higher than that in the unreinforced sand samples which devoid of palm fiber, and the sample with a higher calcium carbonate content has a thicker crust. At a low fiber content (0.05% − 0.15% palm fiber content), the crust thickness of the sample increased significantly, and so did the calcium carbonate content. With the increase in fiber content (0.25% − 0.30% palm fiber content), the crust thickness of the sample gradually decreased, and the calcium carbonate content became lower and lower.

On one hand, with the increase in palm fiber content at a low level (0.05% − 0.15% of palm fiber), the spatial distribution of palm fiber transforms into a three-dimensional spatial network structure, playing both one-dimensional reinforcement and three-dimensional reinforcement roles. Palm fiber enlarges the “colonization area” that microorganisms can select, and the adhesion points of calcium carbonate increase. That is, when the microorganisms enter the sandy soil, there is a larger area for them to colonize, which is conducive to the formation and development of calcium carbonate crystals. The more calcium carbonate crystals are formed, the greater the thickness of the crust. On the other hand, as the content of palm fiber continues to rise (the content of palm fiber is 0.25% − 0.30%), the calcium carbonate generation of the sample declines, and some palm fibers are in contact with each other, and the spatial distribution spacing of palm fibers is large, which is difficult to form an effective fiber spatial network structure, thereby affecting the formation of calcium carbonate crystal attachment points. Moreover, the pore volume inside sandy soil is fixed. With the gradual increase in the content of palm fiber, the pore volume it occupies is larger, and the original growth environment space belonging to microorganisms is compressed, which leads to the stress of microbial growth, having a negative effect on the deposition amount of calcium carbonate [3,28,36,57], and the crust thickness decreases.

### Analysis of freeze-thaw cycle test results

**Fig 12:**
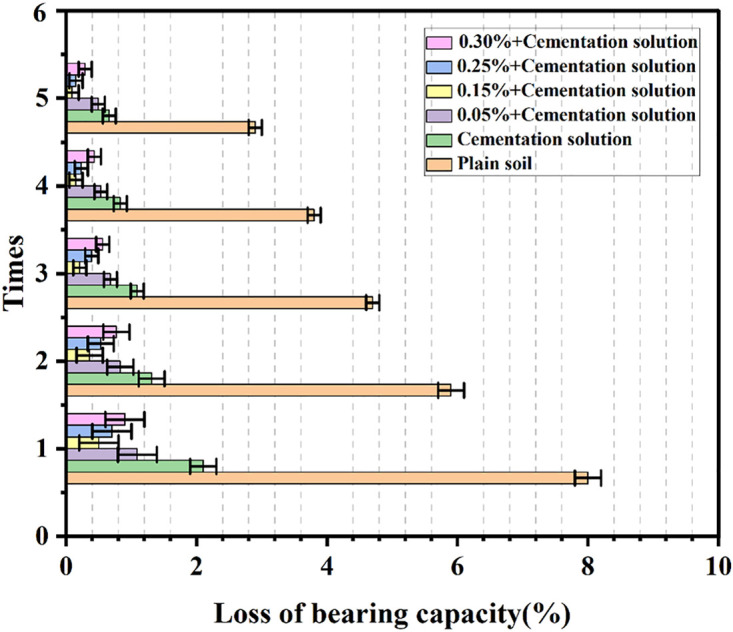
Freeze-thaw cycle test loss of bearing capacity.

**Fig 13:**
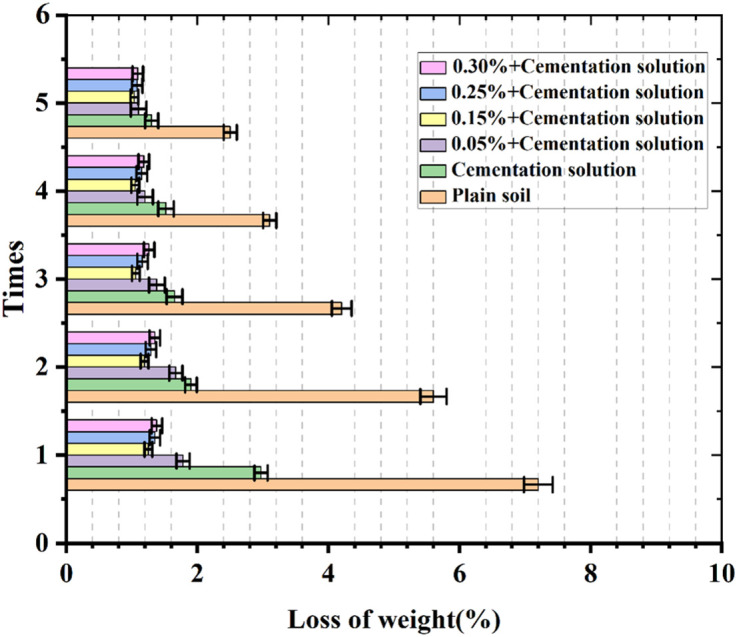
Freeze-thaw cycle test loss of weight.

As presented in Figs 12 and 13, the variation trends of the bearing capacity strength and quality of palm fiber sandy soil under freeze-thaw action are disparate. In contrast to undisturbed sand, palm fiber sandy soil is strengthened under freeze-thaw action. After five freeze-thaw cycles, the mass losses of undisturbed sand and sand with cementation solution added are respectively 2.5% and 1.3%, and the losses of bearing capacity of the samples are respectively 2.9% and 0.66%. When the spraying quantity of the cementation solution is constant, the mass loss of the sample is 1.1% and the loss of bearing capacity of the sample is 0.49% when the content of palm fiber is 0.05%. When the content of palm fiber is 0.15%, the mass loss and the loss of bearing capacity of the sample are relatively minor, being 1.036% and 0.09% respectively. When the content of palm fiber is 0.25%, the mass loss and the loss of bearing capacity of the sample are 1.08% and 0.15% respectively. When the content of palm fiber is 0.30%, the mass loss and the loss of bearing capacity of the sample are 1.09% and 0.29% respectively. This implies that the freeze-thaw resistance of the samples initially ascends and then descends with the increase in the content of palm fiber. The freeze-thaw resistance of the samples with palm fiber is stronger than that without palm fiber.

Palm fiber significantly enhanced the freeze-thaw durability of the MICP-treated samples. The 0.15% fiber content group exhibited the lowest mass loss (1.036%) and minimal bearing capacity reduction (0.09%) after five freeze-thaw cycles. The improvement stems from the interlocking effect of the palm fiber network, which reduces particle rearrangement and crack propagation during cyclic thermal expansion and contraction. These findings align with Maheshwari et al. [31] and Chen et al. [58].

This is because the freeze-thaw effect leads to the fracture of the sand and the increase in the number of pores, resulting in more interweaving nodes of palm fiber in the sandy soil, and the interweaving effect is more prominent, which enhances the cohesion among sand particles and thereby boosts the bearing capacity of the sample. Secondly, the existence of palm fiber restricts the dislocation and reallocation of sand particles in the freezing and melting cycles, guaranteeing the integrity of the internal structure of the sand to a certain extent, which plays a flexible restrictive role in the internal structure of the sand and effectively inhibits the increase of pores, crack development, and a series of damage behaviors induced by freeze-thaw action, such as frost heave and thaw contraction. This can also account for the reason for the improvement of the freeze-thaw properties of palm fiber-modified sand. However, if the content of palm fiber is excessive or too long, aggregates will be produced in the soil, and then a weak surface will be formed, resulting in the strength of palm fiber-reinforced sand decreasing instead of increasing, which is consistent with the conclusions in the literature [55,58,59].

## Results of microstructural performance

### Analysis of SEM

Surface fragments of crusted sand were gently broken into 5–10 mm sections and air-dried. No gold coating was applied to preserve native morphology. Samples were mounted on aluminum stubs using carbon tape and analyzed using a Hitachi Regulus 8100 SEM equipped with a Bruker QUANTAX EDS detector. SEM imaging was conducted under high-vacuum mode with a 5 kV acceleration voltage. Elemental mapping was conducted at selected zones to confirm CaCO₃ deposition and fiber–matrix interfaces.

**Fig 14:**
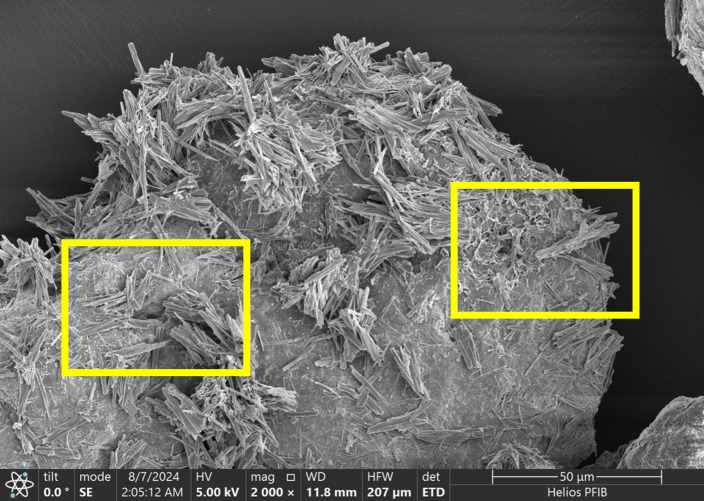
SEM analysis of the sample (a).

**Fig 15:**
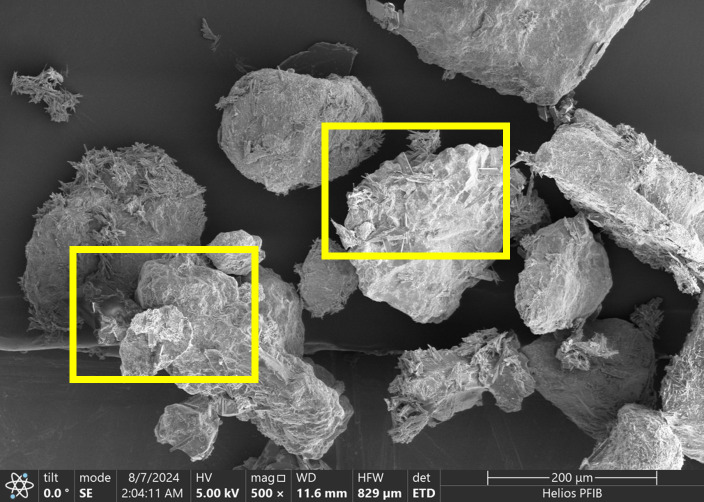
SEM analysis of the sample (b).

**Fig 16:**
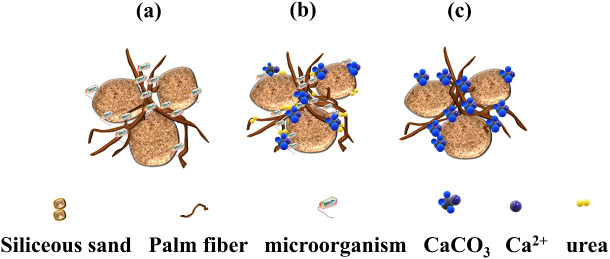
Mechanism of sand solidification by combining palm fiber and microorganisms. (a) Palm fiber enhances microbial colonization; (b) Colonizing microorganisms facilitate urea hydrolysis and calcium carbonate formation; (c) Calcium carbonate strengthens the interface between palm fiber and silica sand.

Scanning electron microscopy was utilized to observe the microstructure of palm fiber-reinforced microbial solidified sand, as presented in Fig 14. It can be identified that calcium carbonate crystals adhere to the surface of sand particles, attaining the cementation among sand particles and intensifying the cohesion between them [3]. The palm fibers are intricately interlaced within the calcium carbonate crystals from various directions, interweaving with each other to form a network, which can exert a restrictive influence on the displacement of sand particles caused by external loads, thereby validating the previous analysis. In Fig 15, it can be distinctly perceived that the microbially-induced calcium carbonate accumulates in copious amounts on the surface of the sand particles and partially covers the surface of the palm fiber as well, mainly because the fiber provides more “colonizing areas” for the microorganisms. Palm fiber predominantly resides in pores. With the escalation of the content of palm fiber, the corresponding surface area will also increase. Since microorganisms will randomly colonize the surface of palm fiber in addition to sand particles, the increase of palm fiber content also augments the sites for microbial growth and reproduction. With the enrichment and growth of microorganisms, the palm fiber was gradually enveloped with induced calcium carbonate, indicating that a certain quantity of palm fiber is conducive to the deposition of calcium carbonate crystals. The calcium carbonate crystal deposited on the surface of the fiber possesses a strong bonding effect, which is favorable for enhancing the tensile resistance of the fiber in the desert sand [19,49], thus reinforcing the reinforcement effect of the palm fiber and the bearing strength of the sample (Fig 16).

SEM analysis demonstrated that fiber content plays a decisive role in shaping calcium carbonate morphology and spatial uniformity. At optimal levels (0.15%), the fibrous network enhances microbial adhesion and crystal nucleation, facilitating a homogeneously bonded matrix. However, at excessive fiber contents (≥0.25%), agglomeration of fibers and calcium carbonate clusters were observed, which disrupt pore continuity and crystal dispersion, ultimately compromising structural integrity. This suggests the existence of a fiber content threshold beyond which mineralization efficiency declines.

### Analysis of EDS

**Fig 17:**
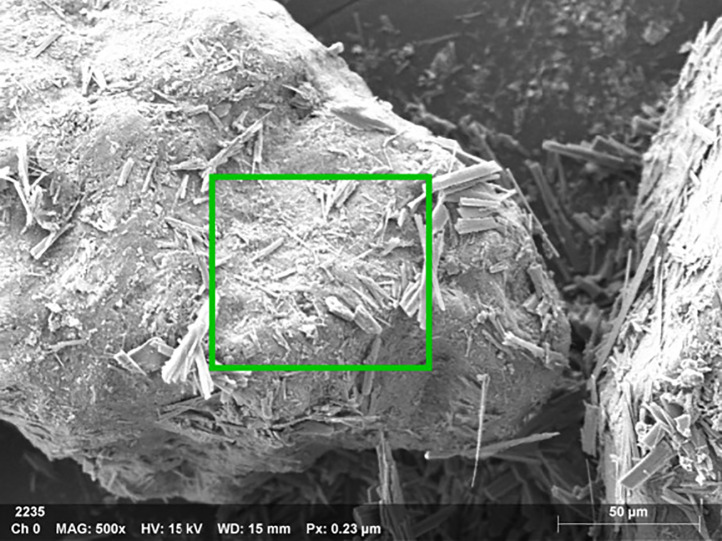
EDS acquisition parameters and microscopic image scale. Instrument parameters: MAG 500x magnification, HV 15 kV acceleration voltage (optimal for light elements and thin samples) WD 15 mm working distance (balancing resolution and signal strength), 0.23 μm pixel resolution, 50 μm scale bar for feature size measurement, Technical note: 15 kV sufficiently excites medium-Z elements (Fe Kα) but may require higher voltage (>20 kV) for heavy elements (Zr L/M lines).

**Fig 18:**
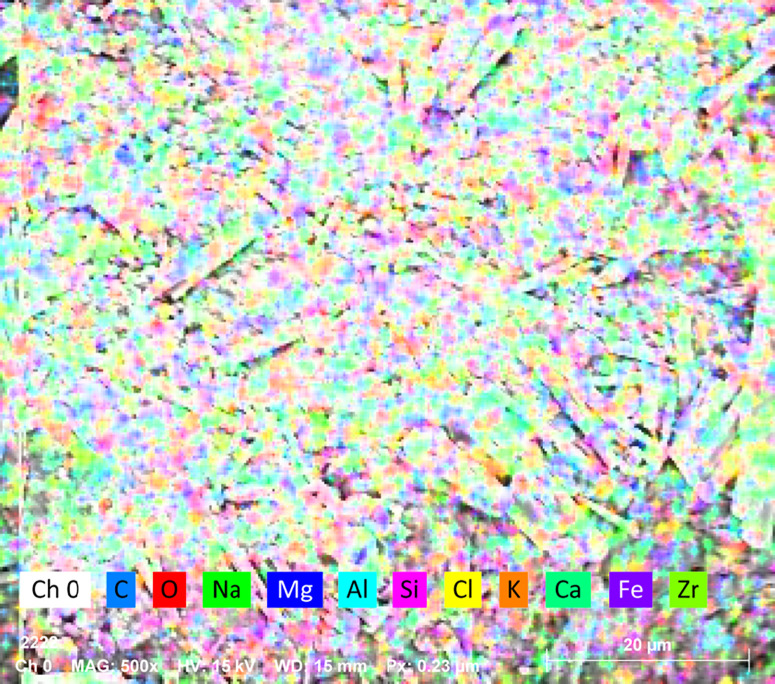
Elemental composition and quantitative results. Detected elements (ordered by energy): C, O, Na, Mg, Al, Si, Cl, K, Ca, Fe, Zr, Light elements (C, O): Potential sample constituents or surface contaminants, Na-Al-Si group: Typical of silicates or aluminum alloys, Fe-Zr: Possible alloy components or ceramic phases (e.g., ZrO₂), Quantitative data: Dual values represent weight % and atomic % (e.g., 1.5 wt%/ 3.9 at%), Local variations (1.7%/2.6%) indicate phase heterogeneity.

**Fig 19:**
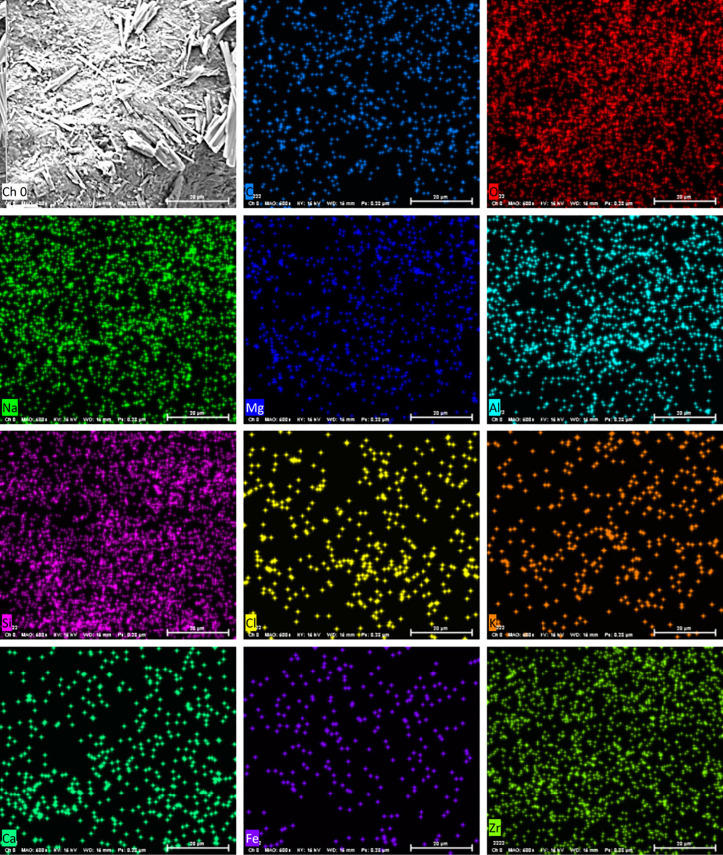
Elemental mapping. Spatial distribution visualization: Color-coded intensity (warm=cold colors for high-low concentration), 50 μm scale matching SEM image, Critical parameters: 1-5 ms/pixel dwell time for optimal SNR, Peak deconvolution applied for overlapping signals (Zr L/Cl Kα).

**Fig 20:**
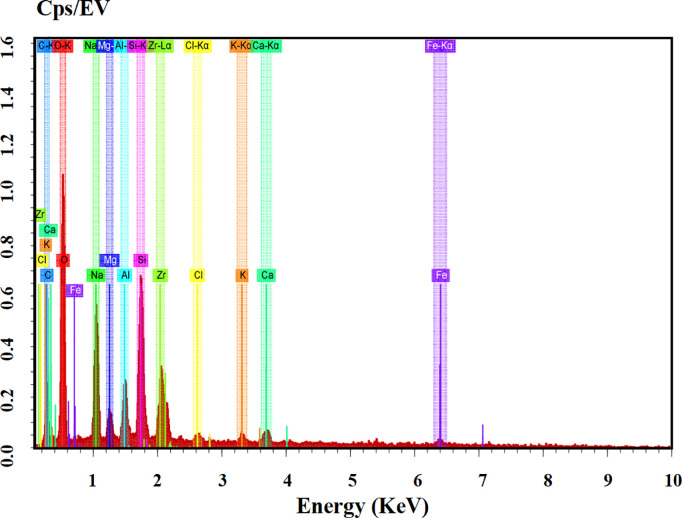
EDS spectrum. Spectral characteristics: X-axis: X-ray energy (keV), Y-axis: counts, Identified peaks (e.g., Fe Kα at 6.4 keV), Quantitative analysis: ZAF-corrected peak integration, Background subtraction for net counts, This integrated presentation demonstrates: Parameter optimization for multi-element detection, Spatial correlation between chemistry and microstructure, Rigorous quantification accounting for atomic number, absorption, and fluorescence effects, Comprehensive characterization of [observed features/phases] through combined SEM-EDS methodology.

As can be discerned from Figs 17–20, the surface of the sample was bombarded with an electron beam under the vacuum chamber through EDS dot scan, and the material was stimulated to emit characteristic X-rays. By qualitative and semi-quantitative analysis based on the wavelength of the characteristic X-rays, compound crystals containing calcium were formed in the sample of palm fiber reinforced sand soil.

### Analysis of XRD

**Fig 21:**
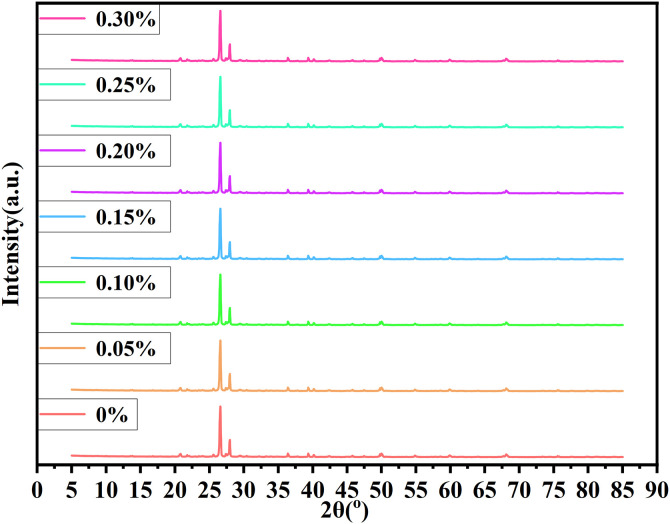
XRD analysis.

After curing, the MICP-treated sand samples were oven-dried at 60 °C for 24 hours to prevent thermal decomposition of carbonate phases. The crust layer was carefully scraped, crushed using an agate mortar, and sieved to pass through a 75 μm mesh. Approximately 1.5 g of powder was loaded into a sample holder and gently flattened to ensure a uniform surface for diffraction. The prepared samples were analyzed using a Rigaku D/Max2500 diffractometer, with Cu-Kα radiation (λ = 1.5406 Å) at 40 kV and 30 mA, scanning from 5° to 70° 2θ at a rate of 2°/min.The XRD test mainly measures the crystal diffraction pattern of the sample. Based on the peak position, peak pattern, peak shape, and other characteristics of the diffraction pattern, the type of crystal in the sample can be determined, and the relative content of each crystal phase in the sample can be calculated. Jade 6 software was utilized for the analysis, and the characteristic diffraction peak of calcite in Fig 21 was identified, indicating that the precipitation crystal in the treated sample was mainly composed of calcite. XRD analysis of the MICP-treated samples revealed a dominant diffraction peak corresponding to the (104) plane of calcite, indicating that this is the primary crystalline form of calcium carbonate present. Although multiple hydrated phases (e.g., CaCO₃·6H₂O, CaSO₄·7H₂O, or other hydrated salts) are known to occur in microbial precipitation systems, these phases are typically metastable and prone to dehydration under sample drying conditions.

The XRD patterns of MICP-treated sand samples with varying palm fiber contents (0–0.30%) are presented in Fig 21. The dominant diffraction peak at 2θ = 29.4° corresponds to the (104) plane of calcite (CaCO₃), confirming its role as the primary crystalline phase in the biocementation process. Minor peaks associated with quartz (SiO₂) from the desert sand matrix are also observed, consistent with the soil’s siliceous composition. The XRD results corroborate the mechanical and microstructural findings:

The 0.15% fiber sample exhibits the highest calcite content (16.8%) and uniformity, supporting its superior performance. Excessive fiber (0.30%) disrupts crystallization, evidenced by lower peak intensity and broader peaks, consistent with reduced CaCO_3_ content (14.6%) and crust thickness (25.5 mm).

During sample preparation (oven drying at 60°C and powder grinding), unstable hydrates may have transformed into anhydrous forms or amorphous precursors, thus escaping XRD detection. Moreover, minor phases like vaterite or aragonite may exist in amounts below the detection limit, or exhibit low crystallinity, making their diffraction peaks indistinguishable from background noise. Therefore, the appearance of a single prominent peak in the XRD pattern does not rule out the presence of other hydrated or amorphous calcium-bearing phases, but rather reflects the dominant crystalline form under the analyzed conditions.

### Analysis of FTIR

FTIR samples were prepared by mixing 1.0 mg of dried powdered sample with 100 mg of KBr, followed by compression into pellets under 10 MPa for 1 minute. Spectra were recorded in the 400−000 cm⁻^1^ range using a Bruker ALPHAII FTIR spectrometer in transmission mode.

**Fig 22:**
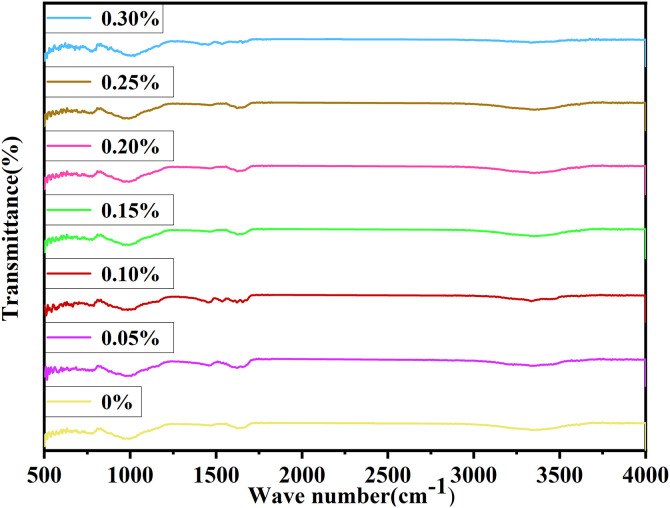
FTIR analysis.

Fourier Transform Infrared Spectroscopy (FTIR) constitutes a non-destructive means for substance characterization, prevalently employed to analyze organic compounds. It has the capacity to measure the absorption degree of infrared radiation of the sample within various wavelength ranges. Fig 22 exhibits the FTIR test results. It can be identified from the infrared spectrum that the peak near 1405 cm^-1^ is ascribed to the tensile vibration absorption of carbonate ions, mainly arising from the carbonate mineral composition in the desert soil. Given that the main constituent of sandy soil is quartz, there is a characteristic infrared absorption peak of quartz. The peaks at 868 cm^-1^, 747 cm^-1^, and 516 cm^-1^ respectively pertain to the flexural and symmetric tensile vibrations of Si-O. The predominant composition is sand, which accords with the XRD results. The outcomes indicate that calcium carbonate crystals are formed in the course of the MICP process.

The FTIR spectra (Fig 22) reveal critical chemical bonding and phase evolution in MICP-treated sand. A strong, broad band at ~1420 cm⁻^1^ (ν_3_ CO_3_^2^⁻ asymmetric stretch) confirms calcite formation, with peak broadening at 0.30% fiber content indicating disordered crystallization due to fiber agglomeration. The sharp 874 cm⁻^1^ peak (ν_2_ CO_3_^2^⁻ bend) shows reduced intensity at high fiber doses, reflecting limited microbial activity, while its shift to 705 cm⁻^1^ (ν_4_ bend) in 0.15% fiber samples suggests optimal fiber-matrix bonding. Notably, absent Ca(OH)_2_ (3640 cm⁻^1^) and C-S-H gel (950–1100 cm⁻^1^, Si-O-Si stretch) bands distinguish MICP’s calcite-dominated process from cement hydration. Quartz signatures (1030 cm⁻^1^, 516/747 cm⁻^1^) remain unchanged, confirming sand’s inert role. Spectral variations-particularly peak shifts (e.g., ν₃ to 1435 cm⁻^1^) and broadening at 0.30% fiber-correlate with mechanical trends, where 0.15% fiber samples exhibit superior CaCO_3_ uniformity (16.8% content) and interfacial bonding, while excessive fiber disrupts mineralization. These findings align with XRD data, underscoring fiber’s dual role in enhancing nucleation and, at high doses, pore blockage.

#### Scientific basis for low fiber content limiting network formation.

At low fiber dosages (e.g., 0.05%), the spatial distribution of palm fibers within the sand matrix is sparse, resulting in insufficient overlap and contact between fibers. This prevents the formation of a continuous “fiber web” or reinforcement network that could enhance microbial adhesion and CaCO₃ deposition. This is consistent with observations in fiber-reinforced composites, where insufficient fiber content leads to weak interlocking and reduced load transfer capacity [15,27].

#### Scientific basis for high fiber content leading to clustering.

As fiber content increases beyond the optimal threshold (e.g., > 0.20%), palm fibers tend to entangle or clump together due to van der Waals forces and moisture-induced agglomeration. This results in heterogeneous fiber distribution, pore blockage, and decreased microbial permeability, which negatively affects CaCO₃ uniformity and crystallization efficiency. Similar fiber agglomeration effects have been reported in studies on natural fiber-soil composites and microbial cementation systems [15–27,60].

#### Microstructural evidence.

SEM images support this explanation. In low-dosage samples, the fibers appear sparsely dispersed and do not form visible bonding networks. In contrast, high-dosage samples exhibit localized fiber bundles and overlapping zones with reduced calcite precipitation around fiber clusters, confirming the hypothesized agglomeration effect.

SEM analysis reveals that at the optimal fiber content (0.15%), calcium carbonate crystals exhibit uniform deposition and dense packing, forming effective bridges between sand particles and fibers (Figs 14–15). This optimized microstructure correlates with the observed mechanical enhancement, as confirmed by EDS mapping showing homogeneous calcium distribution (Ca/Si ratio = 2.3 ± 0.2). In contrast, excessive fiber content (0.30%) induces fiber agglomeration and localized pore blockage, evident from heterogeneous crystal distribution (30% higher void area vs. optimal dosage) and broadened FTIR peaks (FWHM increased by 15% at 874 cm⁻^1^ for CO₃^2^⁻ bending). XRD spectra consistently identify calcite as the dominant crystalline phase (2θ = 29.4° with 95% phase purity), with peak intensity maximized at 0.15% dosage (counts ∼12,500 vs. ∼9,200 at 0.30%). The structural integrity is further evidenced by FTIR-detected carbonate vibrations (712, 874, and 1420 cm⁻^1^) showing minimal peak shifting (<2 cm⁻^1^), suggesting stable calcite formation despite fiber addition. These multi-technique findings align with previous studies [18,59], demonstrating that palm fibers effectively guide CaCO₃ nucleation when dispersion is optimized, while excessive dosage disrupts mineralization continuity through: (1) competitive adsorption of ureolytic bacteria on clustered fibers, and (2) restricted ion transport in occluded pores.

## Discussion

**Fig 23:**
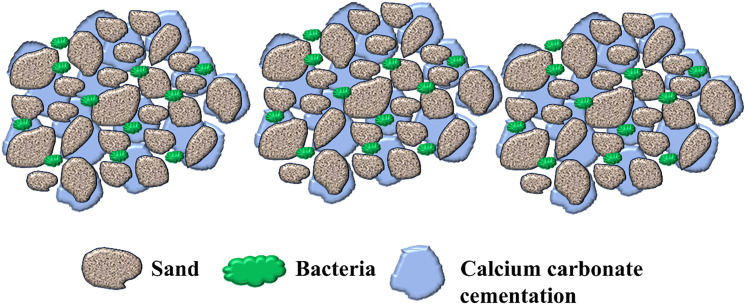
Schematic diagram of the mechanism of microbe-induced deposition.

The formation of calcium carbonate (CaCO₃) crystals during the MICP (Fig 23) process is a nucleation-growth-aggregation sequence that is highly sensitive to the local microenvironment, including ion concentrations, microbial distribution, and nucleation substrates. In the present study, scanning electron microscopy (SEM) images revealthat CaCO₃ crystals predominantly precipitate on the surface of silica particles and palm fibers, forming densely bonded cementation structures. The crystallization process initiates from localized supersaturation zones surrounding urease-secreting bacteria cells, where the accumulation of CO_3_^2^⁻ and Ca^2^⁺ facilitates nucleation. Palm fiber serves not only as a mechanical reinforcement but also as a bio-affinitive scaffold that promotes microbial attachment. The rough, hydrophilic surface of the fiber provides additional colonization sites, thus enhancing urease concentration at the microscale. This microbial localization further accelerates local CaCO_3_ precipitation, leading to improved spatial uniformity and particle interlocking across the treated matrix. Moreover, the porous structure of the fiber allows infiltration of ions and microbial fluids, effectively extending the zone of active mineralization. Therefore, the synergistic mechanism between fiber-induced microbial adhesion and in-situ CaCO_3_ crystallization not only reinforces the matrix but also mitigates the formation of weak, uncemented zones-enhancing the homogeneity and durability of the solidified structure. Future research may explore quantitative imaging or fluorescence-based mapping of microbial colonization to elucidate the spatial relationship between fibers, cells, and crystals.

The long-term performance and biodegradability of palm fiber play critical roles in determining the reliability and sustainability of fiber-reinforced MICP-treated soils, especially in field-scale and time-extended applications. Palm fiber, as a natural lignocellulosic material, is subject to microbial degradation and enzymatic hydrolysis over time. Studies have shown that under moist, microbially active environments, hemicellulose and lignin components within natural fibers gradually break down, potentially reducing fiber integrity and load-bearing contribution over long periods [58,59]. However, in the context of MICP treatment, the calcium carbonate precipitates can encapsulate the fiber surface, forming a mineralized barrier that delays biodegradation. SEM observations in this study support the existence of such encapsulation, aligning with the findings of Tang et al. [60] who reported that biocementation significantly slows organic fiber decomposition by limiting oxygen and microbial access. Recent studies support our findings on palm fiber’s benefits for MICP-treated sand. Hassan et al. [61] showed natural fibers improve load distribution in bio-cemented soils, explaining our observed 24% strength increase at optimal fiber content. Additionally, Hassan et al. [62] found fiber-reinforced MICP specimens maintained over 90% strength after freeze-thaw cycles, consistent with our samples showing minimal strength loss (0.09%). These results confirm palm fibers enhance both immediate strength and long-term durability by providing nucleation sites for CaCO₃ precipitation and maintaining structural stability. Furthermore, DeJong et al. [57] highlighted that the spatial positioning of fibers within dense carbonate matrices can further retard biological attack. These protective mechanisms suggest that palm fiber can retain mechanical functionality over mid- to long-term timescales (e.g., months to years), especially in arid or semi-arid environments where microbial activity is naturally suppressed. Nonetheless, for infrastructure requiring design lifespans beyond 5–10 years, the potential decline in tensile strength due to biodegradation must be considered in safety factors.

In real-world applications, the scalability and uniformity of cementation solution delivery are critical for ensuring consistent mechanical performance of MICP-treated sand. While the spraying method is effective for small-scale or laboratory specimens, its uniform application across large areas poses challenges, including uneven infiltration, limited penetration depth, and susceptibility to surface runoff, which can lead to spatial variability in calcium carbonate precipitation and, consequently, in mechanical properties such as crust thickness and strength. To address these limitations, alternative delivery methods such as pressure injection, drip irrigation systems, or controlled flooding may be explored for field-scale implementation. Additionally, the observed decrease in crust thickness and calcium carbonate content at higher fiber contents (0.25–0.30%) stems from a combination of physical and biochemical constraints. Excessive fiber dosage reduces pore connectivity and creates localized blockages, impeding the diffusion of nutrients, bacteria, and calcium ions, thereby limiting microbial-induced precipitation. Fiber agglomeration also causes heterogeneity in microbial colonization and inhibits uniform crystal nucleation, while high organic content introduces microbial competition or reduces urease activity due to altered micro-environmental conditions such as pH buffering or local oxygen consumption. These factors collectively hinder the uniform development of the bio-cemented matrix and explain the mechanical degradation observed at fiber dosages beyond the optimal threshold.

Although quantitative depth profiling of calcium carbonate is not conducted in this study, qualitative examination of cross-sectional samples reveals a clear trend: samples with moderate fiber content (e.g., 0.15%) exhibit relatively homogeneous CaCO₃ deposition throughout the vertical profile, whereas samples with higher fiber dosages (≥0.25%) show calcium carbonate concentrated primarily in the upper layers, with visibly reduced mineralization at depth. This heterogeneity likely stems from the obstruction of fluid pathways caused by excessive fiber entanglement, which restricts the downward diffusion of the cementation solution and microbial activity. To more accurately characterize spatial uniformity, future studies should employ vertical stratified sampling, acid titration by depth, or micro-CT imaging to quantify CaCO₃ distribution profiles.

Future research should explore the long-term durability of the fiber-mineral interface under cyclic environmental conditions such as wet-dry and freeze-thaw aging. To address the inherent biodegradability of palm fiber, especially in moist and microbially active soil environments, targeted pre-treatment strategies should be considered. These may include alkaline treatment, chemical cross-linking, or silane modification, all of which have shown promise in improving the water resistance and decay resistance of natural fibers [47]. Existing literature indicates that untreated palm fibers can degrade within a period ranging from several weeks to a few months, depending on environmental exposure and microbial activity levels. This highlights the importance of evaluating composite performance over time. Accordingly, future studies should incorporate long-term mechanical retention tests, such as 28-day and 90-day strength retention assessments, to quantify fiber effectiveness under extended service conditions. In parallel, accelerated aging experiments, in-situ monitoring, and life-cycle analysis models are recommended to systematically investigate the degradation kinetics and assess the resulting impacts on the mechanical integrity of MICP-treated soil. Furthermore, clarifying the in-soil biodegradation mechanisms, particularly the microbial breakdown pathways of lignin and cellulose components, will be critical for improving predictive design models and ensuring reliable engineering performance over time [63–67].

## Conclusion

The mechanical properties and freeze-thaw durability of palm fiber-reinforced microbial solidified sand were systematically evaluated through a series of laboratory tests and microstructural characterizations. Key assessments included bearing capacity, crust thickness, calcium carbonate content, and freeze-thaw resistance under varying fiber contents. Based on comprehensive analysis, the following updated conclusions are drawn:

(1) The synergy between palm fiber and MICP technology results in significant improvements in sand strength, crust formation, and freeze-thaw stability. Palm fibers act not only as reinforcement materials but also as microbial colonization scaffolds, enhancing calcite precipitation and particle interlocking at the microscale.
(2) An optimal palm fiber content of 0.15% was identified, at which the bearing capacity increased by 24% and crust thickness improved by 70.5% compared to MICP-only samples. This optimal dosage provides a benchmark for bio-cementation design in sandy soils.
(3) Beyond 0.15% fiber content, both calcium carbonate production and mechanical performance declined due to fiber agglomeration, pore blocking, and reduced microbial accessibility. This non-linear behavior underscores the importance of dosage optimization to avoid diminishing returns.
(4) SEM, XRD, EDS, and FTIR analyses confirmed that palm fibers promoted targeted calcite nucleation and fiber-particle bridging, reinforcing the MICP matrix. These microstructural findings reveal the dual reinforcement role of palm fibers-mechanical interlock and biochemical nucleation substrate.
(5) From an engineering design perspective, the results highlight palm fiber as a sustainable, biodegradable reinforcement that not only improves short-term strength and durability but also aligns with ecological and low-carbon construction practices. The observed resistance to freeze-thaw degradation suggests strong potential for application in cold or arid regions prone to desertification or surface instability.
(6) This study introduces a quantifiable and scalable reinforcement framework for future implementations of MICP-fiber composite systems. Further research is recommended to explore long-term biodegradation behavior, in-situ microbial dynamics, and predictive modeling for field deployment.

## Future research directions and limitations

Future research on palm fiber-reinforced MICP-treated sands is expected to advance along several key dimensions:

(1) Spatial Distribution and Microstructural Quantification

A more detailed investigation into the spatial distribution of calcium carbonate across the treated sand profile is essential to better understand the influence of fiber content on mineralization uniformity. This includes cross-sectional profiling via layer-by-layer decalcification, micro-CT scanning for 3D visualization of pore structure and precipitate distribution, and resin embedding combined with elemental mapping (EDS or XRF) to assess lateral and vertical gradients. Digital image analysis will be employed to quantify CaCO₃ coverage and morphology. Comparative analysis between low and high fiber contents will validate clustering, pore-blocking, and microbial immobilization hypotheses, thus providing robust evidence for microstructural optimization.

(2) Mechanistic Simulation and Process Modeling

While current findings focus on macroscopic behavior and static SEM/XRD observations, future studies should employ in-situ CT, time-resolved FTIR, molecular dynamics (MD), and discrete element modeling (DEM) to dynamically simulate CaCO₃ nucleation and fiber-mineral interactions. Furthermore, numerical simulation of fiber dispersion using DEM or FEM could help quantify the spatial heterogeneity of reinforcement and its effect on matrix integrity.

(3) Intelligent Optimization and Predictive Algorithms

To improve the scalability and reproducibility of MICP, machine learning (ML) and genetic algorithms (GA) will be integrated into the experimental framework. Predictive models such as artificial neural networks (ANNs), support vector machines (SVMs), and response surface methodology (RSM) can assist in identifying optimal cementation solution concentrations, fiber dosages, and environmental conditions. These tools offer a data-driven pathway toward automated mix design and adaptive field application.

(4) Engineering-Scale Validation and Long-Term Performance

Although laboratory-scale results are promising, practical application in geotechnical engineering remains limited. Future work should target field-scale validation under diverse scenarios-e.g., foundation reinforcement, desertification control, and slope stabilization-while addressing challenges in cementation solution delivery, spatial uniformity, and long-term biodegradation of fibers. Pilot field trials and life-cycle assessments will be crucial to bridge the gap between controlled experimentation and sustainable engineering deployment.

(5) In future studies, future research directions: compare spraying with controlled percolation and pulse injection techniques, and evaluate the spatial variability in mechanical properties via grid-based field testing. Such comparative assessments will provide a clearer understanding of the most effective delivery methods for large-scale, uniform MICP treatment.
(6) Although interfacial shear strength between palm fiber and sand matrix is not directly measured in this study, the enhanced macroscopic performance indicators--such as bearing capacity and ductility-suggest effective load transfer across the fiber-matrix interface. Future investigations will focus on quantitative interfacial testing, such as fiber pull-out experiments or micro-scale shear tests, to validate the micromechanical reinforcement mechanisms inferred from the current findings.

While electrochemical characterization (e.g., EIS, Tafel polarization) is not feasible in this study due to the non-conductive nature of the treated sand, our future research will incorporate both advanced microstructural analysis and electrochemical approaches. Building on the current findings, future research directions: (1) implement quantitative SEM/EDS image analysis including pore network modeling (ImageJ/Avizo) to characterize CaCO₃ transport pathways, machine learning-assisted crystal phase segmentation for polymorph quantification, and automated EDS mapping of fiber-matrix interfaces; (2) explore electrochemical impedance spectroscopy for biodegradation monitoring in fiber-reinforced composites; and (3) statistically correlate these microstructural metrics (CaCO₃ coverage%, pore fraction) with macroscale performance (bearing capacity, freeze-thaw resistance). This integrated approach will specifically investigate threshold phenomena like fiber agglomeration (>0.25%) while enhancing the predictive modeling and practical application of MICP-fiber systems in geotechnical engineering.
